# Influencing Factors of Pine Wood Milling Force Based on Principal Component Analysis and Multiple Linear Regression

**DOI:** 10.3390/ma19020439

**Published:** 2026-01-22

**Authors:** Bo Shen, Dietrich Buck, Ziyi Yuan, Zhaolong Zhu

**Affiliations:** 1 Co-Innovation Center of Efficient Processing and Utilization of Forest Resources, Nanjing Forestry University, Nanjing 210037, China; 2 College of Furnishings and Industrial Design, Nanjing Forestry University, Nanjing 210037, China; 3 Wood Science and Engineering, Luleå University of Technology, 931 87 Skellefteå, Sweden

**Keywords:** principal component analysis (PCA), multiple linear regression (MLR), solid wood processing, cutting tool, wood cutting

## Abstract

Milling force is a parameter affecting wood processing quality, tool life, and energy consumption, and its variation is influenced by the multi-factor coupling of cutting parameters and tool geometric factors. This study systematically investigates milling forces during the processing of pine wood (*Pinus sylvestris* var. *mongholica* Litv.) using a hybrid modeling approach combining principal component analysis (PCA) and multiple linear regression (MLR). Firstly, PCA was employed to reduce the dimensionality of the tool rake angle (*γ*), helix angle (*λ*), cutting depth (*h*), feed per tooth (*U*_z_), and triaxial milling forces (*F*_x_, *F*_y_, *F*_z_); this eliminated the multicollinearity among variables and extracted the integrated features. Subsequently, an MLR model was constructed using the principal components as independent variables to quantitatively evaluate the contribution of each factor to milling forces. The results support the conclusion that PCA successfully extracted the first four principal components (cumulative variance contribution rate: 92.78%), with PC1 (49.16%) characterizing the comprehensive milling force effect and PC2 (15.03%) primarily reflecting the characteristics of the tool geometric parameters. The established MLR model demonstrated a high significance (*R*^2^: *F*_x_ = 0.915, *F*_y_ = 0.907, *F*_z_ = 0.852). The cutting depth exerted a significant positive driving effect on the triaxial milling forces via PC1 (each 1 mm increase in depth increased the PC1 score by 0.64 units, resulting in increases of 27.2%, 26.6%, and 21.8% for *F*_x_, *F*_y_, and *F*_z_, respectively). The helix angle significantly suppressed *F*_y_ through PC2 (*β* = −0.090, *p* < 0.001), whereas the rake angle exhibited a weak negative effect on *F*_x_ via PC3 (*β* = −0.015). Parameter optimization identified the combination *γ* = 25°, *λ* = 30°, *h* = 0.5 mm, and *U*_z_ = 0.1 mm∙z^−1^ as optimal, which reduced the triaxial milling forces by 62.3% compared to the experimental maximum. This study provides a theoretical foundation and novel parameter optimization strategy for the efficient, low-damage processing of wood materials.

## 1. Introduction

As a renewable engineering material, wood finds extensive application in construction, furniture, and composite materials [1,2,3,4]. Milling is a common method of shaping wood for these applications. In the milling process, the milling force directly influences the machined surface quality, tool wear, and energy efficiency [5,6,7,8,9]. Pine wood (genus *Pinus*, family *Pinaceae*), like many softwoods, is characterized by its loose texture and significant anisotropy, making it prone to defects like tears and burrs during milling. Consequently, research into its cutting mechanics is vital for process optimization [10,11,12].

The existing research indicates that milling forces are affected by the multi-factor coupling of variables, including wood species, cutting parameters (spindle speed, cutting depth, and feed per tooth), and tool geometric parameters (rake angle, clearance angle, and helix angle) [13,14,15,16,17,18]. Eyma et al. found that cutting forces in the transition zone of Scots pine were 15–20% lower than in earlywood or latewood. Accounting for this observation improved the density–cutting force model accuracy from *R*^2^ = 0.41 to 0.92, which highlighted the need for the separate evaluation of the transition zone in heterogeneous wood processing [10]. In their review, Wei et al. showed that, in wood–plastic composite milling, CrCN/CrN- and ta-C (tetrahedral amorphous carbon)-coated tools exhibited a fivefold increase in tool life, and tools with a 65° rake angle combined with a large helix angle optimized both the surface quality and chip evacuation efficiency [19]. It should be noted that the milling mechanics of solid wood, a naturally anisotropic material, differ from those of more homogeneous engineered composites like MDF. This anisotropy introduces additional complexity in force prediction. Sedlecky et al. demonstrated that the milling energy consumption of thermally modified wood decreased with a reduced wood density (9% reduction after a 210 °C modification), and the energy consumption increased by 166% when the cutting speed rose from 20 m/s to 40 m/s. The optimal energy-saving parameters were a cutting speed of 20 m/s, rake angle of 25°, and feed speed of 4 m/min [20]. Curti et al. proposed a generalized cutting force model for peripheral wood milling that covers the full range of grain orientations (0–360°), by milling circular specimens and quantifying the coupled effects of density, uncut chip cross-section, grain orientation, and tool helix angle [21]. Jiang et al. optimized walnut straight-tooth milling using response surface methodology and found that increasing the rake angle simultaneously reduced the cutting force and surface roughness. The optimal parameter combination was a rake angle of 15°, cutting depth of 0.2 mm, and cutting speed of 45 m/s, with a validation error of less than 5.6% [22].

Traditional single-factor experimental methods struggle to resolve interaction effects, and conventional regression models are susceptible to variable multicollinearity [23]. Although principal component analysis (PCA) has been used for multivariate decoupling in machining fields like metal cutting, its application in wood milling remains limited [24]. In soft materials like pine, elasto-plastic deformation leads to highly nonlinear relationships between force and parameters, necessitating the development of more robust analytical frameworks [25,26,27].

To address these challenges, this paper proposes a hybrid modeling strategy integrating PCA and multiple linear regression (MLR) that aims to resolve the multi-factor coupling problem in pine milling and provide a theoretical basis and optimization pathway for efficient, low-damage pine processing [28,29,30,31]. Therefore, the primary objective of this study is to employ a PCA-MLR hybrid approach to decouple the multi-factor interactions and quantify the individual and coupled effects of the tool geometry and cutting parameters on the triaxial milling forces of pine wood. We hypothesize that PCA will effectively extract dominant, uncorrelated process features, upon which MLR can build robust predictive models to identify the optimal low-force machining parameters.

## 2. Materials and Methods

### 2.1. Experimental Equipment

**Figure 1:**
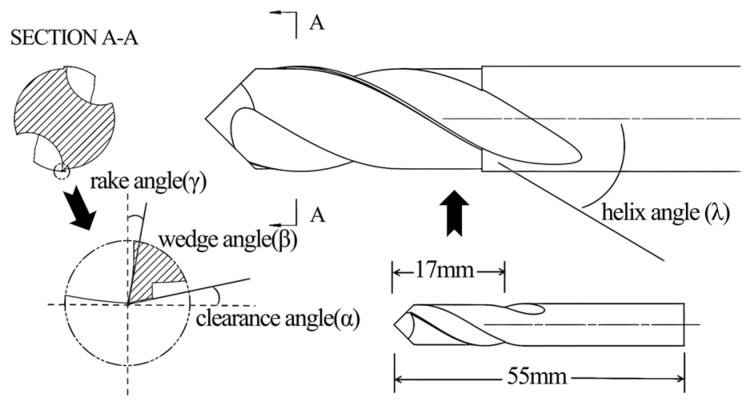
Testing tool structure diagram.

**Figure 2:**
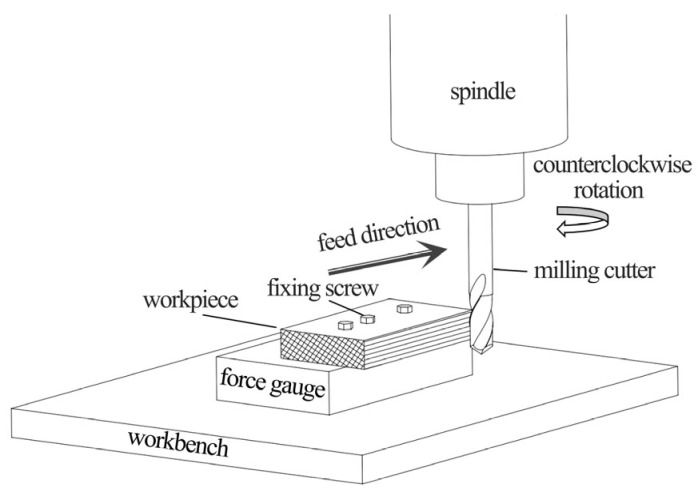
Milling system setup.

The material used was Mongolian Scots pine (*Pinus sylvestris* var. *mongholica* Litv.). obtained from kiln-dried, defect-free sawn timber. The average moisture content (MC) of the workpieces was 10–12%. Workpiece dimensions were 100 mm × 150 mm × 8 mm (length × width × thickness), with a basic density of 0.42 g/cm^3^. The grain orientation was aligned parallel to the length direction (150 mm), which corresponded to the feed direction during milling. To ensure homogeneity across all experimental runs, all samples were selected from the same batch of timber and the surfaces to be milled were lightly pre-planed to achieve a consistent starting condition. The test tools, end mills with carbide tool bodies, were produced by Leuco Precision Tool Co., Ltd. (Taicang, China). Nine tools with different specifications, labeled (a) to (i), were used (Table 1). The rake angle (*γ*) and helix angle (*λ*) encompassed three levels each, whereas clearance angle (*α*) was consistently 15°. Each tool had a total length of 55 mm, milling length of 17 mm, diameter of 8 mm, and two teeth. Tool structure is shown in Figure 1. All tools were right-handed. Milling was performed on a Nanxing MGK01 machining center with an HSK-F63 tool holder interface. The spindle was an 8.1 kW high-speed routing electric spindle, with a maximum speed of 24,000 RPM. The collet chuck accommodated shank diameters from 6 to 20 mm, and the maximum feed speed was 50 m/min. The experimental milling system is shown in Figure 2.

### 2.2. Experimental Design and Procedure

**Figure 3:**
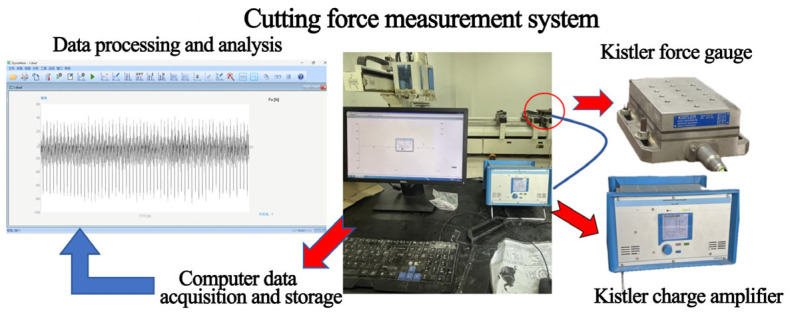
Cutting force measurement system.

Triaxial milling forces (*F*_x_, *F*_y_, and *F*_z_) were measured during milling using a piezoelectric Kistler 9257B triaxial dynamometer (Kistler Group, Winterthur, Switzerland) coupled with a 5070A charge amplifier and corresponding data acquisition and processing system. This voltage-based dynamometer measures forces in three directions with high sensitivity and minimal deformation. The dynamometer was mounted with its coordinate system aligned to the machine tool axes: the *x*-axis corresponded to the feed direction (dominantly reflecting the tangential cutting force component), the *y*-axis was perpendicular to the feed direction in the horizontal plane (dominantly reflecting the radial force component), and the *z*-axis was vertical (axial direction). The forces *F*_x_, *F*_y_, and *F*_z_ reported are the instantaneous resultant forces measured in these fixed machine coordinates, which dynamically integrate the tangential and radial force components throughout the tool rotation. Key technical parameters were sensitivity 0.05 N, range ±5 kN, and stiffness 1 μm/N. The cutting force measurement system is shown in Figure 3. Key factors influencing milling force—tool rake angle (*γ*), helix angle (*λ*), milling depth (*h*), and feed per tooth (*U*_z_)—were each set at three levels within ranges commonly used in pine wood milling, following a full factorial design (Table 2). Rake angles were 15°, 20°, and 25°; helix angles were 10°, 20°, and 30°; milling depths were 0.5 mm, 1.25 mm, and 2.0 mm; and feed per tooth values were 0.1 mm∙z^−1^, 0.25 mm∙z^−1^, and 0.4 mm∙z^−1^. Spindle speed was fixed at 12,000 RPM, and milling direction was up-milling (conventional milling).

Following the statistical requirements of a full factorial design, 81 experimental runs were conducted (3 levels × 4 factors). PCA was performed using Python 3.1.3. Subsequently, MLR prediction models for the milling force components (*F*_x_, *F*_y_, and *F*_z_) were built based on the PCA results. Table 3 presents the experimental data.

#### 2.2.1. Principal Component Analysis (PCA)

The raw dataset comprised 7 variables (rake angle (*γ*), helix angle (*λ*), cutting depth (*h*), feed speed (*U*_z_), and triaxial milling forces (*F*_x_, *F*_y_, and *F*_z_)) with a sample size of *n* = 81. Systematic data preprocessing was performed first. To validate the dataset’s suitability for PCA, the Kaiser–Meyer–Olkin (KMO) measure of sampling adequacy and Bartlett’s test of sphericity were systematically conducted. For further verification, the anti-image correlation matrix was calculated. Finally, eigenvalues, variance explained, and principal component (PC) loadings were computed for the preprocessed data [32].

(1) Systematic Data Preprocessing

Outlier Handling: Potential outliers were detected using Z-score standardization. For each observation *x_ij_* (row *i*, column *j*), the Z-score was calculated as *z_ij_* = (*x_ij_* − *μ_j_*)/*σ_j_*, where *μ_j_* and *σ_j_* are the mean and standard deviation of variable *j*. Observations exceeding the threshold (|*Z_ij_*| > 2.4) were replaced by the median of the corresponding variable *X_j_* [33]:

(1) $$x_{ij}\;=\;\left\{\begin{array}{ll} \mathrm{median}(X_{j}) & \mathrm{if}\;|z_{ij}|\;>\;2.4\\ x_{ij} & \mathrm{otherwise} \end{array}\;\right.$$

with *i* = 1, 2, …, 81, observations and *j* = 1, 2, …, 7 variables.

Logarithmic Transformation: All variables underwent log(*x* + 10^−6^) transformation to improve data distribution characteristics [34].

Standardization: Z-score standardization was applied to eliminate scale effects [35]:

(2) $$z_{ij}\;=\;\frac{x_{ij}-\mu_{j}}{\sigma_{j}},\;i\;=\;1,2,\ldots ,n;\;\;j\;=\;1,2,\ldots ,p$$

where *μ_j_* is the mean of variable *j* and *σ_j_* is its standard deviation.

(2) KMO Test

The KMO test assesses the suitability of a dataset for factor analysis by comparing the magnitudes of simple correlations to partial correlations between variables [36]:

(3) $$\;\mathrm{KMO}\;=\;\frac{\sum \sum_{j\;\neq \;k}r_{jk}^{2}}{\sum \sum_{j\;\neq \;k}r_{jk}^{2}+\;\sum \sum_{j\;\neq \;k}p_{jk}^{2}}$$

where *r_jk_* is the simple correlation between variables *j* and *k*, and *p_jk_* is their partial correlation coefficient. KMO values range between [0,1], and their interpretation is given in Table 4.

(3) Bartlett’s Test of Sphericity

Bartlett’s test verifies if the correlation matrix is an identity matrix (indicating variable independence). The null hypothesis is as follows [37]:

$$H_{0}:\;\mathrm{The}\;\mathrm{correlation}\;\mathrm{matrix}\;R\;=\;I_{p}$$

where **R** denotes the (7 × 7) sample correlation matrix of the standardized variables, **I***_p_* is the (7 × 7) identity matrix, and (*p* = 7) represents the number of variables. The test statistic is as follows:

(4) $$\chi^{2}\;=\;-\left[n-1-\frac{2p\;+\;5}{6}\right]\ln(|R\left|\right)$$

where *n* is the sample size (*n* = 81), *p* is the number of variables (*p* = 7), and |**R**| is the determinant of the correlation matrix.

(4) Anti-Image Correlation Matrix Test

The anti-image correlation matrix is a core tool in factor analysis for assessing variable suitability. It comprises two parts [38]:

- Anti-Image Covariance Matrix:

(5) $$C={\;D}^{-1}(I-{\;R}^{-1})D^{-1}$$

where:

*R* is the variable correlation matrix;

*D* is a diagonal matrix with elements, *d_ij_* = $\sqrt{r^{ij}}$, where *r^ij^* is the diagonal element of *R*^−1^;

*I* is the identity matrix.

b. Anti-Image Correlation Matrix:

(6) $$A=\left[\begin{array}{c} a_{ij} \end{array}\right],\;a_{ij}=\frac{c_{ij}}{\sqrt{c_{ii}\cdot c_{jj}}}$$

The diagonal elements *a_ij_* are the measure of sampling adequacy (MSA) values. MSA*_i_* > 0.5 indicates variable *i* is suitable for factor analysis. Off-diagonal elements reflect partial correlations; values close to 0 indicate high variable independence.

(5) Principal Component Calculation

Eigenvalues *λ_k_* and corresponding eigenvectors **v***_k_* were calculated for the standardized data correlation matrix **R**_p×p_ [39]:

(7) $$R\;=\;\left[\begin{array}{ccc} r_{11} & \ldots & r_{1p}\\ \vdots & \ddots & \vdots \\ r_{p1} & \ldots & r_{pp} \end{array}\right],\;r_{ij}\;=\;\frac{\mathrm{cov}(Z_{i},Z_{j})}{\sigma_{Z_{i}}\sigma_{Z_{j}}}$$

(8) $$(R-\lambda_{k}I)v_{k\;}=0$$

where *Z_i_* and *Z_j_* are standardized variables, *p* = 7 is the number of variables, *λ_k_* is the *k*-th eigenvalue ordered descending as *λ*_1_ ≥ *λ*_2_ ≥ … ≥ *λ_p_*, and **v***_k_* is the corresponding eigenvector satisfying |**v***_k_*| = 1. The variance explained by the k-th principal component is as follows:

(9) $$\mathrm{Variance}\;\mathrm{explained}\;k\;=\;\frac{\lambda_{k}}{{\sum i\;=\;1}^{p}\lambda_{i}}\;\times \;100\%$$

Principal component loadings *l_kj_* of component PC*_k_*on the original variable *X_j_* are as follows:

(10) $$l_{kj}\;={\;v}_{kj}\sqrt{\lambda_{k}}$$

where *v_kj_* is the *j*-th component of the *k*-th eigenvector.

#### 2.2.2. PCA-MLR Milling Force Prediction Modeling

**Figure 4:**
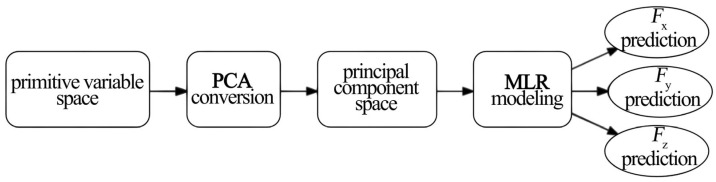
PCA-MLR modeling workflow.

PCA was used to eliminate multicollinearity among original variables and construct an orthogonal feature space. MLR models were then built based on the principal component scores [40]. Figure 4 shows the modeling workflow.

Based on the PCA results, the first *k* principal components satisfying *λ_k_* > 1 and cumulative variance explained ≥ 85% were retained. The principal component score matrix **S***_n_*_×*k*_ (*n* samples, *k* retained PCs) was generated. Independent prediction models for the triaxial milling forces were established using the PC scores *S* as independent variables:

(11) $$\begin{array}{c} F\;=\;f(S)\;+\;\epsilon \end{array}$$

The milling force prediction model in the principal component space is as follows:

(12) $$\hat{F}\;={\;\beta }_{0}\;+{\;\beta }_{1}PC_{1}\;+\;\beta_{2}PC_{2\;}+\;\cdots \;+\;\beta_{k}PC_{k}\;+\;\epsilon$$

where:

$\hat{F}\;=\;[{\hat{F}}_{x},\;{\hat{F}}_{y},{\hat{\;F}}_{z}{]}^{T}$ is the predicted milling force vector;

*PC_k_* = [*PC_k_*_1_, *PC_k_*_2_, …, *PC_kn_*]^T^ is the *k*-th principal component vector;

*β*_0_ is the intercept vector;

*β_k_* is the vector of regression coefficients;

ϵ is the error term vector, assumed ϵ ~ *N*(0,Σ).

Regression coefficients were estimated using ordinary least squares (OLS):

(13) $$\hat{\beta }\;=\;(P^{T}P{)}^{-1}P^{T}F\;$$

where:

P = [1, *PC*_1_, *PC*_2_, …, *PC_k_*] is the principal component design matrix;

F = [*F*_x_, *F*_y_, *F*_z_] is the observed milling force matrix.

## 3. Results and Discussion

### 3.1. Principal Component Analysis

#### 3.1.1. Combined Diagnostic Conclusions

During preprocessing, one outlier was detected and replaced with the median in each of *F*_x_, *F*_y_, and *F*_z_. The KMO and Bartlett’s test results on the preprocessed data are shown in Table 5. The KMO value of 0.702 exceeds the 0.7 threshold, indicating adequate sampling. Bartlett’s test was significant (*p* < 0.001), rejecting the null hypothesis of variable independence. Combined, the tests indicate the dataset is suitable for PCA.

#### 3.1.2. Anti-Image Correlation Matrix Test

To further verify the variable independence, the anti-image correlation matrix diagonal elements (MSA values) were calculated (Table 6). Although the MSA value for the rake angle (*γ*) was low (0.222), considering the overall KMO statistic and significant Bartlett’s test, the data were deemed suitable for PCA as a whole.

#### 3.1.3. Analysis of Eigenvalues and Explained Variance

**Figure 5:**
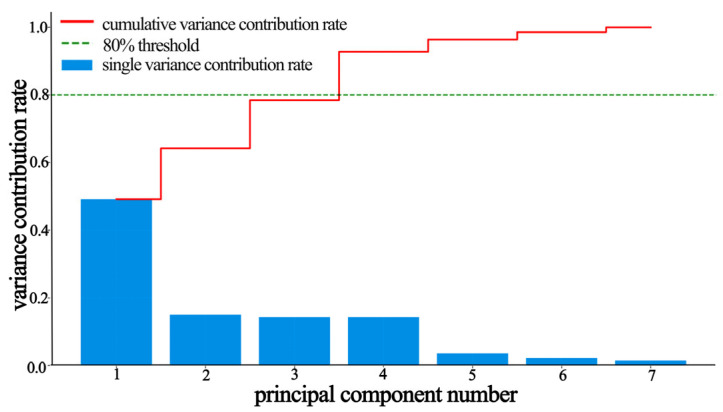
Individual and cumulative variance explained by principal components.

**Figure 6:**
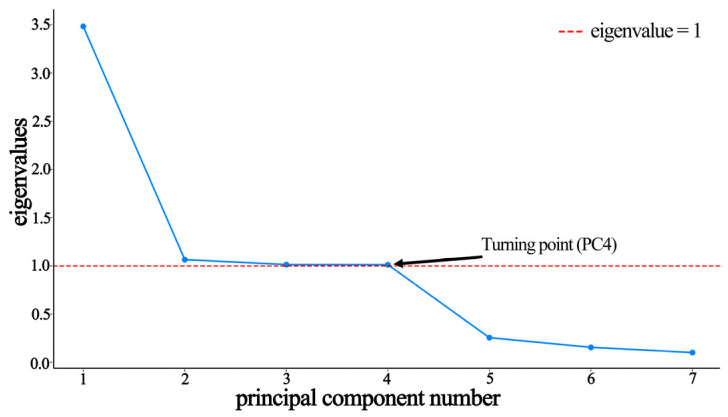
Scree plot of principal component eigenvalues.

The PCA yielded eigenvalues and quantified the variance explained by each principal component (Table 7, and Figure 5 and Figure 6). The first four principal components (PC1–PC4) cumulatively explained 92.78% of the variance. PC1 contributed the most (49.16%), followed by PC2 (15.03%), with PC3 and PC4 contributing similarly (14.30% and 14.28%). Based on Kaiser’s criterion (eigenvalue > 1), the scree plot inflection point after PC4, and cumulative variance > 90%, the first four PCs were retained for a subsequent analysis.

#### 3.1.4. Principal Component Loadings and Physical Interpretation

**Figure 7:**
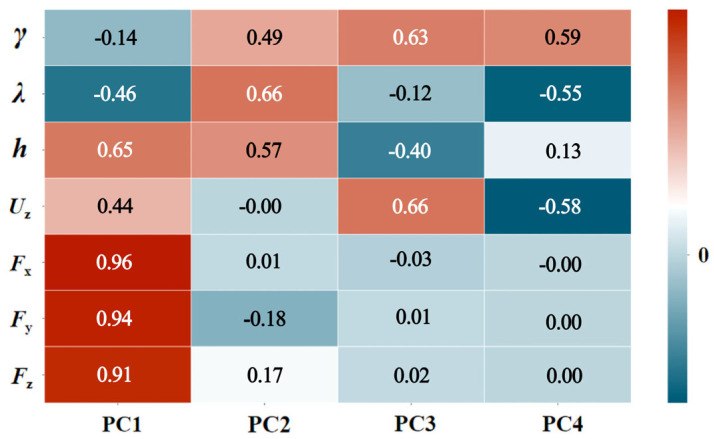
Principal component loadings heatmap.

Table 8 and Figure 7 present the PC1–PC4 loadings matrix. The engineering significance of each principal component is interpreted as follows:

PC1 (Process Intensity Factor): The high positive loadings are on *F*_x_ (0.962), *F*_y_ (0.942), *F*_z_ (0.913), and cutting depth (0.646). The negative loadings are on the rake angle (−0.139) and helix angle (−0.457). PC1 primarily reflects the overall milling force intensity. An increasing cutting depth significantly increases the triaxial forces, whereas an increasing rake or helix angle may reduce the cutting forces.

PC2 (Tool Geometry Factor): The high positive loadings are on the helix angle (0.665) and rake angle (0.487). A weak negative loading is on *F*_y_ (−0.176), and a weak positive loading is on *F*_z_ (0.173). PC2 mainly characterizes the tool geometric parameters, particularly the helix angle. Its variation is negatively correlated with *F*_y_. Although the cutting depth exhibits a notable loading (0.571), its primary influence is captured within PC1. To preserve the consistent interpretation of PC2 as a tool-geometry-dominant factor, it is not highlighted in this discussion.

PC3 (Feed Speed–Rake Angle Interaction Factor): The high positive loadings are on the feed speed (0.664) and rake angle (0.630). PC3 primarily reflects the interaction between the feed speed and rake angle.

PC4 (Tool Geometry Compensation Factor): A high positive loading is on the rake angle (0.595). The high negative loadings are on the helix angle (−0.552) and feed speed (−0.580). PC4 reveals a compensatory or antagonistic relationship between the rake angle, helix angle, and feed speed.

**Figure 8:**
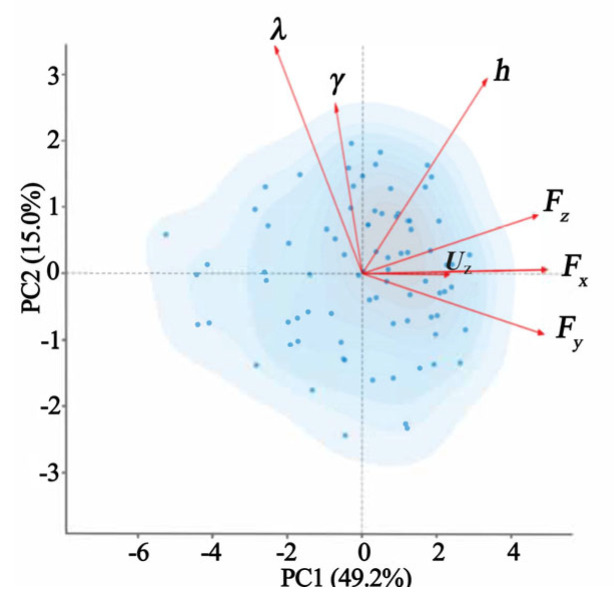
Biplot of PC1 vs. PC2.

Biplots (Figure 8) visually confirm these interpretations by showing the sample distribution and variable loading vectors in reduced spaces. The original variables *F*_z_, *F*_x_, and *F*_y_ cluster along the *x*-axis, which shows a dominant contribution to PC1. The clustering of the original variables helix angle, rake angle, and cutting depth near the *y*-axis indicates their dominant and coordinated contribution to PC2. The distribution of sample points shows a clear trend of increasing dispersion along the cutting depth vector as the PC1 score increases. The biplot for PC3 and PC4, which offered limited additional insight into the primary force-generation mechanisms, has been omitted to sharpen the analytical focus.

It is worth noting that the analysis acknowledges the coupled influence of the cutting depth (*h*) and feed per tooth (*U*_z_) on the uncut chip geometry (e.g., thickness). Rather than attempting to isolate a singular ‘chip thickness’ effect, the full factorial experimental design and subsequent PCA are designed to evaluate the combined and individual contributions of these two parameters to the milling forces. The resulting principal components (e.g., PC1) capture the dominant shared effect, while the loadings in the PC matrix indicate the relative contribution of each original variable to that effect.

Furthermore, in the up-milling configuration with the grain orientation parallel to the feed direction (0°), increasing the cutting depth alters the effective engagement angle between the cutting edge and the wood grain along the cutting arc. This change in grain interaction is an intrinsic part of the cutting process under varying depths. Its effect on cutting forces is inherently captured in the experimental force data and is consequently reflected in the strong association between the cutting depth parameter (*h*) and the milling forces, as extracted by the PCA (primarily in PC1). Thus, the model implicitly accounts for this interaction through the empirical relationship derived from the data.

### 3.2. MLR Model Evaluation and Robustness Analysis

#### 3.2.1. MLR Model Evaluation

The MLR models were built in log-space using the first four PCs. Data preprocessing included outlier handling, log-transformation, and standardization. The model performance was further validated using fivefold cross-validation repeated five times (5 × 5 CV). Table 9 presents the model evaluation.

The PCA-MLR hybrid model performed excellently in predicting triaxial milling forces (Table 9). The *F*_x_ model achieved an *R*^2^ of 0.915 in log-space, indicating PCs captured 91.5% of the force signal variation. When transformed back to the original space, *R*^2^ remained high (0.877) with root mean square error (RMSE) = 5.199 N and mean absolute error (MAE) = 3.777 N, implying the prediction errors averaged below 4 N. The *F*_y_ model achieved a log-space *R*^2^ of 0.907, effectively explaining 90.7% of the lateral force variation (original space *R*^2^ = 0.818, RMSE = 3.541 N, and MAE = 2.417 N). The *F*_z_ model achieved a log-space *R*^2^ of 0.852 (original space R^2^ = 0.772, RMSE = 3.455 N, and MAE = 2.449 N). The adjusted *R*^2^ values (0.911, 0.902, 0.845) confirm the model explains the vast majority of the milling force variance. The 5 × 5 repeated cross-validation results (mean *R*^2^: *F*_x_ = 0.878, *F*_y_ = 0.858, and *F*_z_ = 0.806) demonstrate model robustness and a good generalization ability.

**Figure 9:**
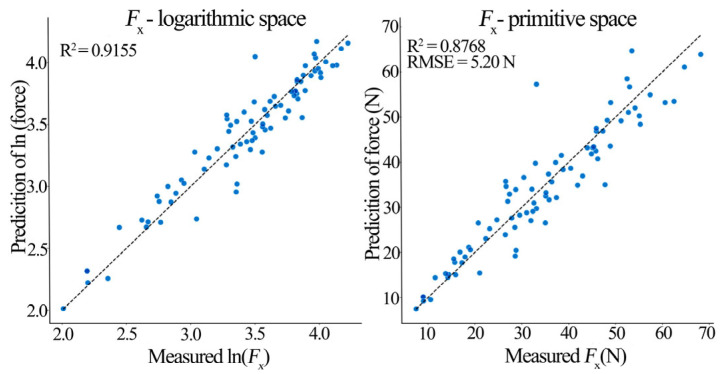
*F*_x_ model prediction performance (Log-space vs. Original-space).

**Figure 10:**
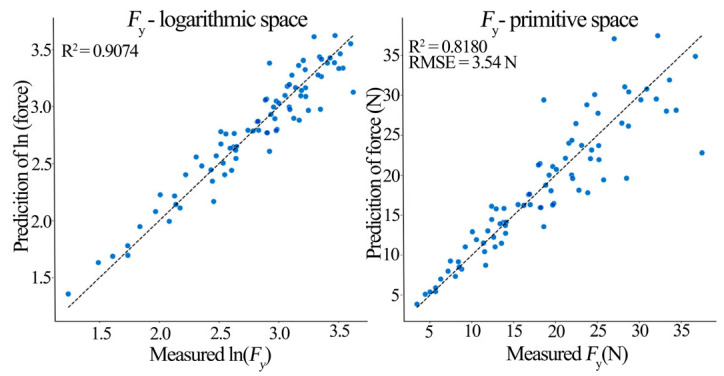
*F*_y_ model prediction performance (Log-space vs. Original-space).

**Figure 11:**
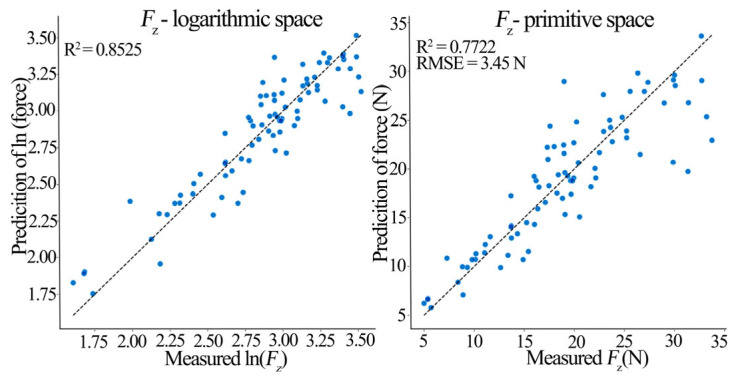
*F*_z_ model prediction performance (Log-space vs. Original-space).

Figure 9, Figure 10 and Figure 11 show prediction performance plots comparing the log-space and original-space models. An analysis of these prediction plots and the MLR coefficients in Table 9 reveals the following insights about the principal components:

PC1 (Process Intensity Factor) had the largest, and highly significant (*p* < 0.001) positive regression coefficient in all three models (*β*_PC1_: *F*_x_ = 0.267, *F*_y_ = 0.266, and *F*_z_ = 0.218). A 1-unit increase in PC1 increased the predicted *F*_x_, *F*_y_, and *F*_z_ by 26.7%, 26.6%, and 21.8%, respectively.

PC2 (Tool Geometry Factor) had differential effects: a significant negative effect on *F*_y_ (*β*_PC2_ = −0.090, *p* < 0.001) indicating it suppresses lateral force, a significant positive effect on *F*_z_ (*β*_PC2_ = 0.075, *p* < 0.001), and no significant effect on *F*_x_.

PC3 (Interaction Factor) and PC4 (Compensation Factor) had non-significant (*p* > 0.05) and near-zero coefficients in all models, indicating a negligible direct contribution to triaxial force prediction, though they represent underlying interactions.

It is noteworthy that the prediction scatter, particularly in the original-space plots, is more pronounced for *F*_y_ and *F*_z_ compared to *F*_x_. This can be attributed to the greater sensitivity of the lateral (*F*_y_) and axial (*F*_z_) force components to the inherent anisotropic and microstructural variability of solid pine wood (e.g., localized density variations, and earlywood/latewood differences), which introduces higher stochastic noise. While this inherent variability presents a challenge for achieving ultra-high precision in absolute force prediction for every individual cut, it does not diminish the model’s primary strength in accurately capturing the dominant trends and facilitating reliable parameter optimization for force reduction, as evidenced by the overall high R^2^ values and successful validation.

#### 3.2.2. Multicollinearity Diagnosis and Assumption Validation

**Figure 12:**
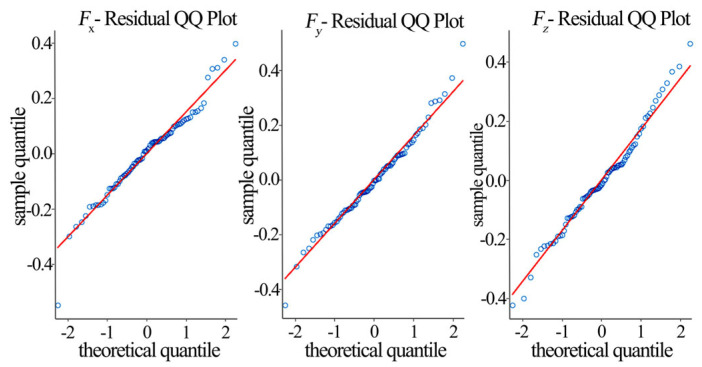
Model residual QQ plots.

**Figure 13:**
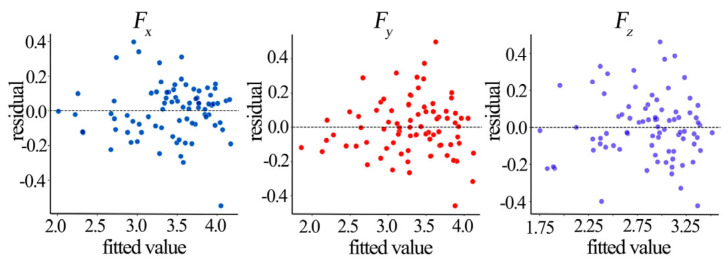
Model residual vs. fitted value plots.

Multicollinearity diagnosis and residual analysis were conducted on the residuals of the established PCA-MLR models using the standardized principal component scores and the observed cutting force data. The variance inflation factor (VIF) for each principal component was 1.0, confirming the absence of multicollinearity among the predictors, which is consistent with the orthogonal nature of the principal components [41]. The residual analysis, including a normal QQ plot and a residual versus fitted values plot, validated the normality and homoscedasticity assumptions of the regression models (Table 10, and Figure 12 and Figure 13).

The variance inflation factor (VIF) values (all 1.0, Table 10) confirm the complete absence of multicollinearity among the selected PC scores. This absence is inherent to the orthogonal transformation that occurs during PCA, which produces uncorrelated PCs. Thus, the regression condition for stable parameter estimates is satisfied.

Residual QQ plots (Figure 12) show points closely aligned with the reference line (an ideal normal distribution), with only a negligible deviation in the extreme tails (±2 theoretical quantiles), which is typical for real datasets. This indicates the residuals for the *F*_x_, *F*_y_, and *F*_z_ prediction models are approximately normally distributed, satisfying the normality assumption for statistical inference. Residual vs. fitted value plots (Figure 13) show no discernible patterns, satisfying the assumption of homoscedasticity.

### 3.3. Analysis of Factor Influence Mechanisms

Cutting Depth Dominance: The depth exerted a significant positive effect (*p* < 0.001) on triaxial forces via PC1. Each 1 mm increase raised the PC1 score by 0.64 units, increasing *F*_x_, *F*_y_, and *F*_z_ by 27.2%, 26.6%, and 21.8%, respectively [42].

Tool Geometry Control: The helix angle increase significantly reduced *F*_y_ (*β* = −0.090, *p* < 0.001) via PC2. likely by improving the chip evacuation efficiency. The rake angle increase interacted with the feed speed via PC3 (loadings 0.630, 0.664). The experimental data showed that, at feed speeds >6000 mm/min, increasing the rake angle to 25° reduced *F*_x_ by 12.6%.

Feed Speed Nonlinearity: Despite the high loading on PC3 (0.664), its MLR coefficient was non-significant, indicating its effect depends on the cutting depth. The experimental data showed that, for depths >1.25 mm, increasing the feed speed to 9600 mm/min increased *F*_z_ by 18.3%.

### 3.4. Cutting Parameter Optimization

#### 3.4.1. Optimization Principle and Method

PC1 (Process Intensity Factor), as the core indicator of the overall cutting load (49.16% variance explained), was minimized to reduce the triaxial milling forces simultaneously. The objective function was defined subject to experimental design space constraints:

(14) $$\min_{\gamma ,\lambda ,h,U_{z}}\mathrm{PC}1\;=\;-0.139\gamma -0.457\lambda \;+\;0.646h\;+\;0.440U_{z}$$

Global optimization algorithms were employed:

(15) $$\left\{\begin{array}{c} 15^{{}^{\circ}}\;\leq \;\gamma \;\leq \;25^{{}^{\circ}}\\ 10^{{}^{\circ}}\;\leq \;\lambda \;\leq \;30^{{}^{\circ}}\\ 0.5\;\mathrm{mm}\;\leq \;h\;\leq \;2.0\;\mathrm{mm}\\ 0.1\;\mathrm{mm}\cdot z^{-1}\;\leq \;U_{z}\;\leq \;0.4\;\mathrm{mm}\cdot z^{-1} \end{array}\right.$$

#### 3.4.2. Optimal Parameter Combination and Experimental Validation

The PC1 minimization criterion yielded the global optimum: *γ* = 25°, *λ* = 30°, *h* = 0.5 mm, and *U*_z_ = 0.1 mm∙z^−1^. The predicted forces at this setting were as follows: *F*_x_ = 7.48 N, *F*_y_ = 3.88 N, and *F*_z_ = 5.78 N, representing a 62.3% reduction compared to the maximum experimental force values. The corresponding experimental measurement (Test Group 73) was *F*_x_ = 7.46 N, *F*_y_ = 3.45 N, and *F*_z_ = 5.68 N. Error analysis (Table 11) shows errors < 2% for *F*_x_ and *F*_z_, validating the model reliability, though the *F*_y_ prediction error was larger (12.46%, possibly related to pine anisotropy affecting lateral force stability).

## 4. Conclusions

This study systematically analyzed the coupled effects of the cutting parameters (depth, and feed per tooth) and tool geometry (rake angle, and helix angle) on the triaxial milling forces (*F*_x_, *F*_y_, and *F*_z_) during the processing of pine wood (*Pinus sylvestris* var. *mongholica* Litv.). The research used a hybrid PCA-MLR modeling approach and proposed optimization strategies. The key conclusions are as follows:

1. The PCA-MLR hybrid model successfully eliminated multicollinearity and decoupled multi-factor interactions. It achieved a high prediction accuracy for the triaxial milling forces, confirmed by robust 5 × 5 repeated cross-validation results, to provide a reliable tool for the high-precision prediction of wood milling forces.
2. The analysis of the principal component physical meaning and MLR models revealed that the PC1 (Process Intensity Factor) is the core driver of milling forces and embodies the strong positive correlation between the cutting depth and triaxial forces. PC2 (Tool Geometry Factor) primarily characterizes the influence of the helix and rake angles, and considerably suppressing lateral force *F*_y_ in the MLR model. The increased helix angle optimizes the chip evacuation, reducing *F*_y_. PC3 (Feed Speed–Rake Angle Interaction) and PC4 (Tool Geometry Compensation) showed a negligible direct contribution to the force prediction but revealed nonlinear parameter interactions.
3. The cutting depth is the strongest positive driver of milling forces, primarily acting through PC1. The helix angle significantly reduces *F*_y_ via PC2. The rake angle increase can weakly suppress *F*_x_ through its interaction with the feed speed (PC3). The feed speed exhibits nonlinear effects dependent on the cutting depth.
4. Based on PCA, PC1 (Process Intensity Factor) minimization yielded the global optimum parameter combination: *γ* = 25°, *λ* = 30°, *h* = 0.5 mm, and *U*_z_ = 0.1 mm∙z^−1^. This combination reduced the predicted milling forces by 62.3% compared to the experimental maximum, validated by low prediction errors for *F*_x_ and *F*_z_.

## Figures and Tables

**Table 1:** Tool angle combinations.

Tool	Rake Angle γ (°)	Clearance Angle α (°)	Helix Angle λ (°)
(a)	25	15	10
(b)	20	15	10
(c)	15	15	10
(d)	25	15	20
(e)	20	15	20
(f)	15	15	20
(g)	25	15	30
(h)	20	15	30
(i)	15	15	30

**Table 2:** Experimental factors and levels.

Level	Rake Angle *γ* (°)	Helix Angle *λ* (°)	Cutting Depth *h* (mm)	Feed per Tooth *U*_z_ (mm∙z^−1^)
1	15	10	0.5	0.1
2	20	20	1.25	0.25
3	25	30	2.0	0.4

**Table 3:** Experimental data.

No.	*γ* (°)	*λ* (°)	*h* (mm)	*U*_z_ (mm∙z^−1^)	*F*_x_ (N)	*F*_y_ (N)	*F*_z_ (N)	No.	*γ* (°)	*λ* (°)	*h* (mm)	*U*_z_ (mm∙z^−1^)	*F*_x_ (N)	*F*_y_ (N)	*F*_z_ (N)
1	15	10	0.5	0.1	31.94	22.79	11.59	42	20	20	1.25	0.4	48.21	28.70	28.96
2	15	10	0.5	0.25	38.44	34.40	22.14	43	20	20	2	0.1	35.11	18.16	16.18
3	15	10	0.5	0.4	46.19	25.07	19.93	44	20	20	2	0.25	60.56	27.92	27.34
4	15	10	1.25	0.1	45.90	33.24	22.50	45	20	20	2	0.4	43.97	24.17	17.56
5	15	10	1.25	0.25	52.73	28.27	22.92	46	20	30	0.5	0.1	8.98	5.00	8.88
6	15	10	1.25	0.4	64.68	32.20	26.98	47	20	30	0.5	0.25	9.03	4.43	5.36
7	15	10	2	0.1	62.46	39.89	33.19	48	20	30	0.5	0.4	19.06	13.53	13.71
8	15	10	2	0.25	54.16	24.68	20.22	49	20	30	1.25	0.1	14.22	6.28	9.30
9	15	10	2	0.4	68.16	27.00	26.33	50	20	30	1.25	0.25	29.55	13.37	18.26
10	15	20	0.5	0.1	11.53	11.65	8.38	51	20	30	1.25	0.4	47.76	16.78	17.36
11	15	20	0.5	0.25	35.02	16.21	13.70	52	20	30	2	0.1	16.82	8.40	14.31
12	15	20	0.5	0.4	20.72	19.79	16.01	53	20	30	2	0.25	32.59	12.37	18.40
13	15	20	1.25	0.1	27.38	23.85	19.67	54	20	30	2	0.4	55.06	23.11	29.89
14	15	20	1.25	0.25	32.16	18.82	17.46	55	25	10	0.5	0.1	17.90	11.39	13.37
15	15	20	1.25	0.4	48.96	30.19	31.38	56	25	10	0.5	0.25	17.24	9.22	9.79
16	15	20	2	0.1	35.82	16.98	13.67	57	25	10	0.5	0.4	23.15	13.84	15.23
17	15	20	2	0.25	53.00	32.03	30.07	58	25	10	1.25	0.1	33.13	12.36	18.81
18	15	20	2	0.4	52.44	30.90	29.99	59	25	10	1.25	0.25	26.56	19.23	22.06
19	15	30	0.5	0.1	10.55	5.68	4.98	60	25	10	1.25	0.4	47.29	22.43	24.77
20	15	30	0.5	0.25	8.98	5.67	5.38	61	25	10	2	0.1	28.71	16.90	19.33
21	15	30	0.5	0.4	15.71	11.53	10.11	62	25	10	2	0.25	37.22	17.96	18.93
22	15	30	1.25	0.1	15.48	7.45	15.41	63	25	10	2	0.4	53.46	36.70	32.67
23	15	30	1.25	0.25	24.69	13.76	16.35	64	25	20	0.5	0.1	14.41	8.79	12.63
24	15	30	1.25	0.4	45.79	21.18	25.22	65	25	20	0.5	0.25	15.90	8.49	8.82
25	15	30	2	0.1	26.65	15.52	31.34	66	25	20	0.5	0.4	36.40	21.92	29.86
26	15	30	2	0.25	45.12	19.67	25.21	67	25	20	1.25	0.1	32.91	28.48	33.73
27	15	30	2	0.4	45.34	18.13	23.61	68	25	20	1.25	0.25	40.43	22.05	23.78
28	20	10	0.5	0.1	18.69	18.60	11.04	69	25	20	1.25	0.4	27.00	19.66	15.97
29	20	10	0.5	0.25	37.38	20.10	17.10	70	25	20	2	0.1	32.36	14.00	16.46
30	20	10	0.5	0.4	42.97	21.59	19.62	71	25	20	2	0.25	35.69	19.46	19.94
31	20	10	1.25	0.1	38.80	25.19	19.05	72	25	20	2	0.4	45.89	24.31	25.56
32	20	10	1.25	0.25	44.85	21.95	18.97	73	25	30	0.5	0.1	7.46	3.45	5.68
33	20	10	1.25	0.4	51.17	23.74	23.52	74	25	30	0.5	0.25	13.74	7.16	14.88
34	20	10	2	0.1	48.88	25.15	17.98	75	25	30	0.5	0.4	22.31	12.61	20.52
35	20	10	2	0.25	57.37	28.74	22.90	76	25	30	1.25	0.1	21.01	8.03	7.27
36	20	10	2	0.4	75.77	33.61	32.72	77	25	30	1.25	0.25	27.86	11.91	21.65
37	20	20	0.5	0.1	28.60	12.78	10.16	78	25	30	1.25	0.4	35.15	12.91	26.56
38	20	20	0.5	0.25	28.78	10.55	11.10	79	25	30	2	0.1	31.03	14.03	19.82
39	20	20	0.5	0.4	26.50	14.10	13.67	80	25	30	2	0.25	41.90	18.25	17.29
40	20	20	1.25	0.1	28.52	10.06	19.07	81	25	30	2	0.4	55.27	37.49	50.26
41	20	20	1.25	0.25	30.42	25.75	20.39								

**Table 4:** KMO test evaluation criteria.

KMO Value	Suitability Assessment
>0.90	Excellent
0.80–0.89	Good
0.70–0.79	Mediocre
0.50–0.69	Fair
<0.50	Unacceptable

**Table 5:** KMO and Bartlett’s test results summary.

Test Metric	Statistic	Reference Standard	Result	Interpretation
KMO Measure	0.702	>0.50	Suitable	Significant partial correlations
$\mathrm{Bartlett}\;\chi^{2}$	357.82	-	-	-
$\mathrm{Degrees}\;\mathrm{of}\;\mathrm{Freedom}\;\left(df\right)$	21	-	-	-
*p*-value	5.92 × 10^−57^	<0.001	Reject H_0_	Correlation matrix not an identity

**Table 6:** Anti-image correlation matrix diagonal elements (MSA values).

Variable	Rake Angle *γ*	Helix Angle *λ*	Cutting Depth *h*	Feed Speed *U*_z_	*F*_x_	*F*_y_	*F*_z_
MSA	0.222	0.478	0.601	0.486	0.767	0.770	0.854

Note: MSA > 0.5 indicates the variable is suitable for factor analysis.

**Table 7:** Principal component eigenvalues and variance explained.

Principal Component	Eigenvalue *λ_k_*	Variance Explained (%)	Cumulative Explained (%)
PC1	3.485	49.16	49.16
PC2	1.065	15.03	64.19
PC3	1.014	14.30	78.49
PC4	1.013	14.28	92.78
PC5	0.255	3.60	96.38
PC6	0.155	2.19	98.57
PC7	0.101	1.42	100.00

**Table 8:** Principal component loadings matrix.

Variable	PC1	PC2	PC3	PC4
Rake Angle (*γ*)	−0.139	0.487	0.630	0.595
Helix Angle (*λ*)	−0.457	0.665	−0.116	−0.552
Cutting Depth (*h*)	0.646	0.571	−0.401	0.133
Feed per Tooth (*U*_z_)	0.440	0.002	0.664	−0.580
*F*_x_	0.962	−0.013	−0.029	≈0
*F*_y_	0.942	−0.176	0.014	≈0
*F*_z_	0.913	0.173	0.018	≈0

**Table 9:** PCA-MLR model regression results.

Dependent Variable	$F_{x}$	$F_{y}$	$F_{z}$
*β*_0_	3.419 ***	2.807 ***	2.858 ***
*β*_PC1_	0.267 ***	0.266 ***	0.218 ***
*β*_PC2_	0.006	−0.90 ***	0.075 ***
*β*_PC3_	−0.015	0.007	0.008
*β*_PC4_	0.00	0.00	0.00
Log-space *R*^2^	0.915	0.907	0.852
Log-space Adjusted *R*^2^	0.911	0.902	0.845
Log-space RMSE (N)	0.151	0.160	0.172
Original-space *R*^2^	0.877	0.818	0.772
RMSE (N)	5.199	3.541	3.455
MAE (N)	3.777	2.417	2.449
5 × 5 Cross-Validation *R*^2^ (Mean ± SD)	0.878 ± 0.092	0.858 ± 0.100	0.806 ± 0.072

Notes: (1) *** *p* < 0.001; (2) *β*_PC4_ coefficients were non-significant (*p* > 0.05) and near zero.

**Table 10:** Multicollinearity diagnosis results (VIF).

Variable	Constant	PC1	PC2	PC3	PC4
VIF	1.0	1.0	1.0	1.0	1.0

**Table 11:** Validation of optimized parameter.

Milling Force	Predicted (N)	Exp. Max (N)	Reduction (%)	Measured (N)	Error (%)
*F*_x_	7.48	75.77	90.1	7.46	+0.27
*F*_y_	3.88	39.89	90.3	3.45	+12.46
*F*_z_	5.78	50.26	88.5	5.68	+1.76
Overall	-	-	62.3	-	-

## Data Availability

The original contributions presented in this study are included in the article. Further inquiries can be directed to the corresponding author.
